# A New Method for Recognizing Cytokines Based on Feature Combination and a Support Vector Machine Classifier

**DOI:** 10.3390/molecules23082008

**Published:** 2018-08-11

**Authors:** Zhe Yang, Juan Wang, Zhida Zheng, Xin Bai

**Affiliations:** School of Computer Science, Inner Mongolia University, Hohhot, Inner Mongolia 010021, China; 15848111501@163.com (Z.Y.); imuzzd@163.com (Z.Z.); 6530071@163.com (X.B.)

**Keywords:** PSSM, PseAAC, SVM, feature combination, cytokines

## Abstract

Research on cytokine recognition is of great significance in the medical field due to the fact cytokines benefit the diagnosis and treatment of diseases, but the current methods for cytokine recognition have many shortcomings, such as low sensitivity and low F-score. Therefore, this paper proposes a new method on the basis of feature combination. The features are extracted from compositions of amino acids, physicochemical properties, secondary structures, and evolutionary information. The classifier used in this paper is SVM. Experiments show that our method is better than other methods in terms of accuracy, sensitivity, specificity, F-score and Matthew’s correlation coefficient.

## 1. Introduction

Cytokines are mainly proteins or peptides generally associated with inflammation and cell differentiation, including interleukins (IL), interferons (IFNs), etc. They can contribute to the diagnosis and treatment of diseases [1,2], for example, they are conducive to the treatment of hematopoietic dysfunction [3], tumor [4], infection [5,6] and inflammation [7,8]. With the post-genomic era coming, the quantity of new proteins has increased dramatically. Some complex experiments based on biology and immunology are required to identify cytokines from these new proteins, which means high time consumption and expensive cost, but the primary structures of these proteins are easier to obtain. In this case, we propose a method which can recognize cytokines quickly and accurately. It is helpful for medical scientists, for instance, the method can reduce the experimental range and workload. They can selectively test the functions for the proteins which are identified as cytokines by this method. In other words, there is no need to test the functions which cannot be related to cytokines. Generally speaking, the method we proposed has two important steps, i.e., the extraction of features and the classifier selection.

There are many algorithms for extracting features from protein sequences. The following will introduce several algorithms for feature extraction. Nakashima et al. [9] first proposed an algorithm which can obtain 20-dimensional features from a protein sequence based on amino acid composition (AAC for short). After that, Luo et al. [10] further extended it to 400 dimensions in terms of the composition information of polypeptide. Based on the physicochemical properties of proteins, Shen et al. proposed the PseAAC algorithm [11]. Besides, several algorithms have been proposed in terms of position-specific score matrices (PSSM) which can be computed by PSI-BLAST software [12]. Additionally, several features [13,14] can be obtained based on structure probability matrices (SPM) and secondary structure sequences (SSS), which can be extracted by PSI-PRED software [15]. The method based on local discriminant bases is used in the latest classification of protein [16] and an algorithm combining PSSM and PseAAC is currently proposed for protein prediction [17].

There are many classifiers for recognizing protein function, such as Rough Set [18], Naive Bayes [19] and SVM [20], of which SVM is very effective. As a supervised learning model, SVM has been widely used in many domains due to its simplicity and efficiency, such as protein subcellular prediction [21], HIV-1 and HIV-2 proteins prediction [22], gene selection [23], protein subcellular localization [24], pre-microRNA prediction [25] and membrane protein function prediction. But the used Data have a serious effect on the efficiency of SVM.

So far, a considerable amount of literature has been published on the prediction of cytokines. Huang et al. used SVM and 7-fold cross-validation to predict and classify the cytokine superfamily [26]. Zeng et al. improved the ability of cytokine prediction by n-gram algorithm and genetic algorithm [27]. Jiang et al. enhanced the performance of cytokine identification by using proper features and classifiers [28]. The advantages of these methods are high accuracy and specificity. However these methods also have some shortcomings, such as low sensitivity and low F-score. For example, the best accuracy in the experiments of Jiang et al. is 93% but the corresponding sensitivity only is 51%.

In this paper, we aimed to improve the performance of cytokines recognition and the efficiency of the SVM classifier when the number of positive and negative sequences is not balanced. Compared with the feature extraction methods in the papers of Huang et al. and Zeng et al., we comprehensively considered four aspects of protein sequences for building a stable model. Meanwhile, compared with the current method, we improved a new method of feature extraction and adopt it in our model. A lot of experiments were done by us to find the optimal feature combination and the hyper-parameter of SVM. Besides, we gave the suggestion for further improvement. We initially obtained 450 features on the basis of composition information and physicochemical properties. Then we extracted structure probability matrices, position-specific score matrices, second structure sequences and selected 418 features from them as a vector. Finally, we selected 448 features from 868 features (450 plus 418) as optimal features and SVM was used as our classifier. The experiments show that our method is superior to other researches by using 10-fold cross-validation methods and an independent test set.

## 2. Classifier and Verification Methods 

SVM is a suitable classifier for protein prediction and classification, such as the prediction of protein secondary structure [29], the prediction of protein interaction [30], protein structure classification [31] and the prediction of protein families [32]. SVM is more applicable to a binary classification problem, which is consistent with our problem. Therefore, we chose SVM to classify protein sequences. It can construct a linear separating hyperplane in high-dimensional feature space to distinguish positive sequences and negative sequences. However, it will be of high computational complexity to compute a hyperplane in high-dimensional space, but SVM can reduce the cost of computation by means of kernel functions. We used the linear kernel function and Gaussian kernel function in our experiments and the LIBSVM [33] software.

10-Fold cross-validation was used to evaluate the generalization ability of our method. In the process of the 10-fold cross-validation, all sequences are randomly divided into ten groups. One of them is used as the test set, and the rest is used as the training set. It should be ensured that each one of ten groups has been tested separately, therefore the evaluated method will be tested ten times in one experiment and there will be ten results. The average of ten results is taken as the final evaluation result. Besides, we also prepared an unbiased independent test set which is randomly selected from the total sample and accounts for the 20% of it. In the test set, the ratio of the positives to the negatives is 1:9, which is the same as that in training set. It can be ensured that the similarity of each sequence between training set and test set is less than 0.6 by the CD-HIT software. We have uploaded the independent test set to GitHub (https://github.com/DeveloperMrYang/FCSVM).

## 3. Measurements

*Acc*, *Sens*, *Spec*, *F*-*score* and *Mcc* were used to measure the performance of our method, and they can be calculated by the following formulas. Here *TP* is the number of true positives, i.e., the number of the positives which are predicted as positives. *FP* is the number of false positives, i.e., the number of the negatives which are predicted as positives. *TN* is the number of true negatives, i.e., the number of the negatives which are predicted as negatives. *FN* is the number of false negatives, i.e., the number of the positives which are predicted as negatives. *Acc* measures the accuracy of the method, which is the ratio of correctly predicted data in all tested data. *Sens* measures the sensitivity of the method, which is the ratio of correctly predicted positives in all tested positives. *Spec* measures the specificity of the method, which is the ratio of correctly predicted negatives in all tested negatives. *Pre* is the ratio of correctly predicted positives in all predicted positives. *F*-*socre* measures the quality of the method by considering *Sens* and *Pre*. *Mcc* is Matthew’s correlation coefficient [34]. It should be noted that *Mcc* is −1 when both *TP* and *FP* are 0 and the model has the poorest predictive ability:

(1) $$Acc=\frac{TP+TN}{TP+TN+FP+FN}\;$$

(2) $$Sens=\frac{TP}{TP+FN}\;$$

(3) $$Spec=\frac{TN}{TN+FP}\;$$

(4) $$Pre=\frac{TP}{TP+FP}\;$$

(5) $$F-score=\frac{2\times Pre\times Sens}{Pre+Sens}\;$$

(6) $$Mcc=\frac{TP\times TN-FP\times FN}{\sqrt{\left(TP+FP)(TP+FN)(TN+FP)(TN+FN\right)}}\;$$

## 4. Feature Combinations and Results

### 4.1. Performance of Feature Methods

We separately tested the performance of four feature extraction methods mentioned above. The classifier used by us was SVM and we used the linear kernel function and the Gaussian kernel function.

Table 1 shows the results of each feature extraction method with different kernel functions. In the experiments, both *TP* and *FP* are 0 when using the n-gram feature method and Gaussian kernel function, therefore the prediction is invalid, which is not listed in Table 1. The results show that the *F_PseAAC_* with Gaussian kernel function performs best in *Acc*, *F*-*score*, and *Mcc*. Besides, in the recognition of negative sequences, the *F_pssm_*_-380_ with Gaussian kernel function is the best because the *Spec* value reaches 93.250%. Correspondingly, in the identification of positive sequences, the *F_n_*_-*gram*_ with linear kernel function performs best and the *Sens* is 85.576%.

### 4.2. Feature Combinations

We combined the *F_pssm_*_-20_ with *F_pssm_*_-380_ to obtain a total of a 400-D feature vector (*F_pssm_* for short), and combined *F_sss_* with *F_PseAAC_* to obtain a 48-D feature vector (*F_sp_* for short). *F_pssm_* was combined with *F_sp_* to obtain a 448-D feature vector (*F_psp_* for short), and finally *F_n_*_-*gram*_ was combined with *F_psp_* to get a 868-D feature vector (*F_pspn_* for short). Table 2 shows the results of feature combination methods with different kernel functions.

From Table 2, we found that the *F_pspn_* with 868 dimensions and linear kernel function performs better in almost all aspects than other vectors. Nevertheless, it should be noted that the dimensions of *F_pspn_* are 420 more than the dimensions of *F_psp_*, but the predictive ability of *F_psp_* with liner kernel function only decreases by 0.2% or so. Thus, considering time cost and the quality of feature combinations comprehensively, we selected *F_psp_* with 448 dimensions as our final feature vector.

A grid search algorithm and 3-flod cross-validation were used to choose the best parameters (*C* and *γ*) for *F_psp_* with Gaussian kernel. *C* and *γ* range from 2^−5^ to 2^5^ with a step size of 2^1^. The accuracy of different *C* and *γ* is shown in Table 3. When *C* is 2^2^ and *γ* is 2^−5^, we get the optimal accuracy.

After parameters optimization, the new result shows that *F_psp_* with Gaussian kernel is better than it with linear kernel and we conducted 10 times 10-fold cross-validation on it. The mean ± standard deviation for each measurement is shown in Table 4. In the final result, the accuracy is 90.84%, the sensitivity is 89.23%, the specificity is 92.42%, the *F*-*score* is 90.63% and the *Mcc* is 81.71%.

### 4.3. Comparison with Other Methods

Jiang et al. [28] downloaded cytokines sequences from the Uniprot database website and used CD-HIT software to process data. Our methods of data acquisition and processing are the same, but the number and proportion of positive and negative sequences we used are different, which will have an effect on the comparison results. In order to eliminate the factor, we randomly divided the 9146 positive sequences into nine groups, then combined positive sequences in each group and all negative sequences into a new dataset. In the new dataset, the number and proportion of positive and negative sequences are consistent with those of Jiang et al., and we did new experiments on the nine datasets to find the optimal feature combination. It follows that *F_psp_* with liner function is better than other feature combinations in almost all metrics except *Spec* from Table 5. From all results, we finally took *F_psp_* with 448 dimensions as our optimal feature vector.

**Figure 1:**
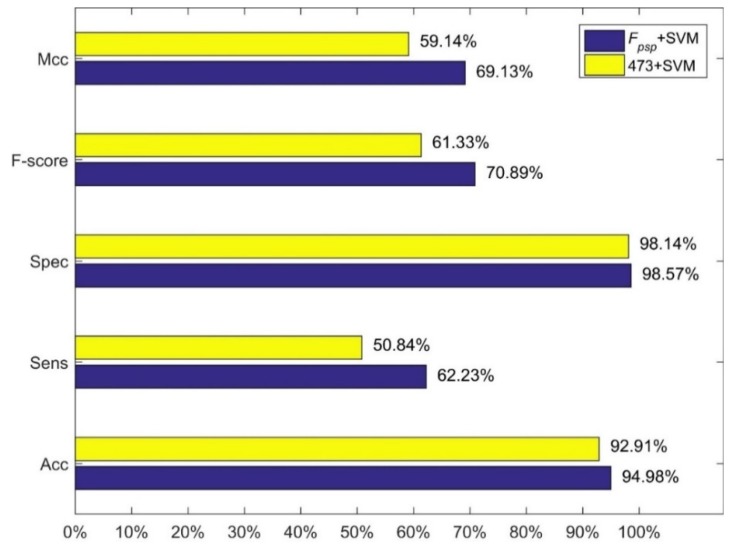
Comparison between our method and the 473+SVM.

**Figure 2:**
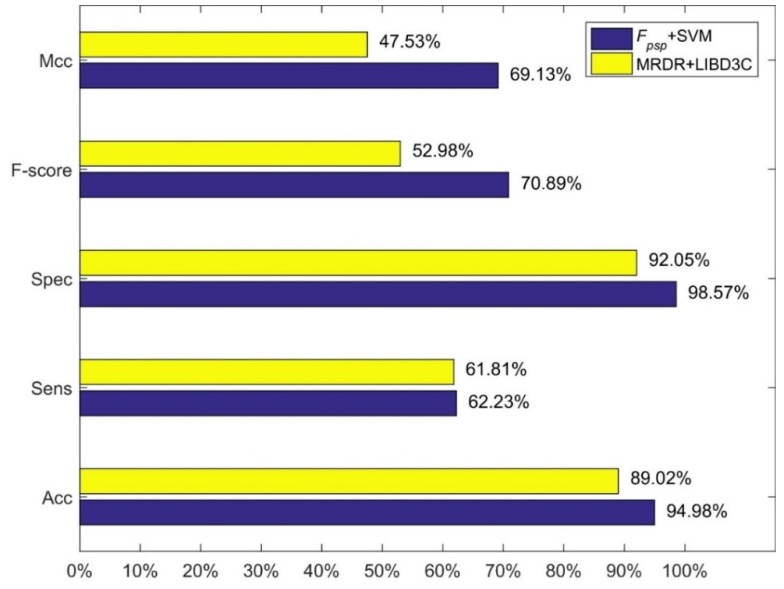
Comparison between our method and the MRDR+LIBD3C.

**Figure 3:**
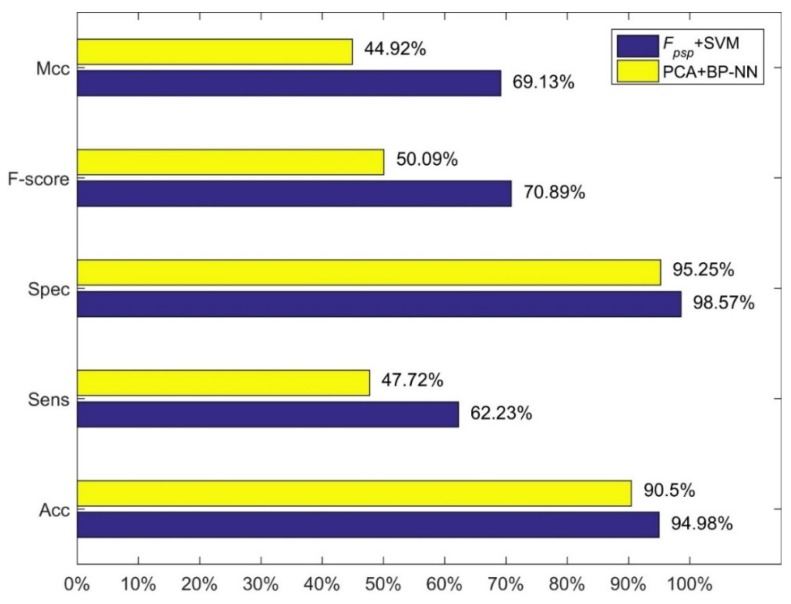
Comparison between our method and the PCA+BP-NN.

We compared our method, i.e., feature combination *F_psp_* with SVM and linear kernel function, with three methods of Jiang et al. The results are shown in Figure 1, Figure 2 and Figure 3. Figure 1 shows the comparison between our method and the method using 473-D feature vector and SVM. Figure 3 shows the comparison between our method and the method using MRDR dimensionality reduction method and the LIBD3C classifier. Figure 3 shows the comparison between our method and the method using PCA dimensionality reduction method and the BP-NN classifier. It is obviously that our method is better than their methods from those figures. In the independent test set we prepared, we also obtain high performance. The accuracy is 93.25% which is also higher than their methods.

## 5. Discussion

Useful information can be extracted from large amount of sequences by appropriate feature extraction methods. Meanwhile, the choice of classifier has an impact on the results of cytokines recognition. In order to obtain higher performance, we focused on the optimal feature combination and adjusted the hyper-parameter appropriately in our experiment, which can promote the characterization of cytokines and build a more appropriate classifier.

Although we achieved higher accuracy, we have some suggestions for further improvement. Firstly, feature selection using sparse regressions [35] like LASSO or elastic net can help further improve the performance and better understand. Secondly, SVM has been widely used in many fields. Thus, we suggest that some deep learning methods should be used in the next experiments, such as deep neural networks with large dataset or the cascade random forests with small dataset. Finally, another sequence-based feature split amino acid composition can also be used in new experiments [36,37].

## 6. Conclusions

We extracted the *F_n_*_-*gram*_ features based on amino acid composition, the *F_PseAAC_* features based on physicochemical properties, the *F_pssm_* features based on evolutionary information and the *F_sss_* features based on secondary structure. Then we used the combinations of the above features, SVM and 10-flod cross-validation method to find the best performing vector for cytokines recognition. Finally we chose *F_psp_* with 448 dimensions as our feature vector and SVM with linear kernel function as our classifier. The experiments show that our method is superior to others. The results show that we get at least a 2.07% and at most a 5.96% increase in accuracy, at least a 0.42% and at most a 14.51% increase in sensitivity, at least a 0.43% and at most a 6.52% increase in specificity, at least a 9.57% and at most a 20.81% increase in *F*-*score*, at least a 9.99% and at most a 24.21% increase in *Mcc*.

## 7. Materials and Methods

### 7.1. Data

A total of 63,811 cytokine sequences were found and downloaded from the Uniprot database website (http://www.uniprot.org/) and used as positive sequences. We got all families of them, and selected the longest sequence from each family of the rest families, i.e., the families after removing the families of positives from all protein families, as a negative sequence. Here we totally obtained 10,118 negative sequences. Then we used the CD-HIT software to process positive sequences and negative sequences respectively in order to remove the highly similar sequences. To make balance of positive sequences and negative sequences in quantity, we set the positive threshold to 0.6 and set the negative threshold to 0.5, then we achieved 9163 positive sequences and 9327 negative sequences. We deleted the sequences which are so long that we can’t obtain their secondary structures, and the sequences less than 20 in length. Because the extraction of some features requires the length of these sequences to be greater than or equal to 20. Finally, we obtained 9146 positive sequences and 9272 negative sequences.

### 7.2. Feature Extraction

In order to identify cytokines efficiently and build a stable model, we comprehensively considered four aspects of protein sequences, which are the composition of amino acids, physicochemical properties, evolutionary information of amino acids and the structure of protein sequences.

**Figure 4:**
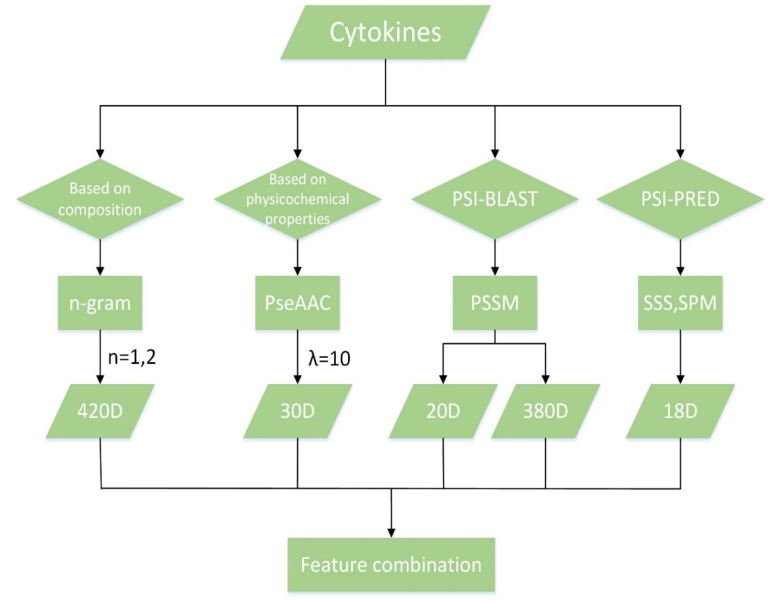
Overview of feature extraction.

We first got 420 features from composition of amino acids by using the n-gram algorithm for *n* = 1 and *n* = 2, and obtained 30 features from physicochemical properties by PseAAC algorithm. Moreover, we computed PSSM by PSI-BLAST software and obtained 400 features about evolutionary information of protein sequences and amino acids. Finally, we extracted secondary structure sequences (SSS for short) and structure probability matrices (SPM for short) by PSI-PRED software to get 18 features about the structure of protein sequences. The process of feature extraction is shown in Figure 4.

#### 7.2.1. *n*-Gram

The n-gram algorithm with a low complexity has been widely used in many fields [38,39]. We obtained features by counting the number of *n* consecutive amino acids in the sequence, where we set *n* = 1 and *n* = 2. Given a protein sequence P, *L* is the length of P and $a_{1}a_{2}\ldots a_{n}$ stands for the spatially adjacent *n* amino acids. For every possible segment $a_{1}a_{2}\ldots a_{n}$ in P, its feature value is computed in detail as follows:

(7) $$F_{n-gram}(a_{1}a_{2}\ldots a_{n})=\left\{\frac{20^{n}}{\sum_{i=1}^{N}20^{i}}\times \frac{T(a_{1}a_{2}\ldots a_{n})}{L-n+1}|n=1,2\cdots N\right\}\;$$

*N* is the maximum value of *n*, where *N* = 2, and $T(a_{1}a_{2}\ldots a_{n})$ represents the number that $a_{1}a_{2}\ldots a_{n}$ appears in P. Therefore, there are 20 features for 1-gram and 400 (i.e., 20^2^) features for 2-gram. Finally, we obtained 420 features by the n-gram algorithm.

#### 7.2.2. PseAAC

However, it is not enough only to take composition information into consideration for cytokines recognition. For example, the amino acid sequences HIDHIHI and HIHIDHI have the same feature vector in the n-gram algorithm, but they are two different sequences because of the different orders of amino acids in the sequences. Then PseAAC algorithm was proposed to solve this problem which is based on composition information, correlation of sequence order and physicochemical properties.

For a protein sequence, a total of 20 + *λ* features can be extracted by PseAAC algorithm and recorded by Formula 2, where $p_{i}$ is the *i*th feature value. The first 20 features are computed based on the composition of the sequence, and other *λ* features describing the correlation of sequence order which are computed in terms of the hydrophilicity, hydrophobicity, and side-chain mass of amino acids. The calculation method is shown in Equation (9) and Equation (10):

(8) $$F_{PseAAC}=\left\{p_{1},p_{2}\ldots p_{20}\ldots p_{20+\lambda }\right\}\;$$

(9) $$p_{n}=\left\{ \begin{array}{l} \frac{f(a_{n})}{\sum_{i=1}^{20}f(a_{i})+w\sum_{j=1}^{\lambda }b_{j}}(1\leq n\leq 20)\\ \frac{wb_{n-20}}{\sum_{i=1}^{20}f(a_{i})+w\sum_{j=1}^{\lambda }b_{j}}(20<n\leq \lambda +20) \end{array}\right.\;$$

(10) $$b_{\lambda }=\frac{\sum_{i=1}^{L-\lambda }\frac{1}{3}\left[{\left(H_{1}(a_{i})-H_{1}(a_{i+}{}_{\lambda })\right)}^{2}+{\left(H_{2}(a_{i})-H_{2}(a_{i+}{}_{\lambda })\right)}^{2}+{\left(M(a_{i})-M(a_{i+}{}_{\lambda })\right)}^{2}\right]}{L-\lambda }\;$$

(11) $$z=\frac{x-\mu }{\sigma }\;$$

Here *λ* expresses the rank of correlation among amino acids of a protein sequence, *w* is the weight factor for the sequence order effect [40], and $a_{i}$ represents the *i*th amino acid in the 20 standard amino acids with the fixed order. $H_{1}(a_{i})$, $H_{2}(a_{i})$, $M(a_{i})$ are the values of hydrophilicity, hydrophobicity and side-chain mass of $a_{i}$, which are calculated by Z-score transformation. The method is shown in Formula 5. *μ* and *σ* represent mean and standard deviation. $f(a_{i})$ is the frequency of $a_{i}$ which is normalized by using Z-score method. PseAAC algorithm has been implemented by PseAAC-Builder software [41,42] and we used the software to obtain a 30-dimensional feature vector when *λ* = 10 and *w* = 0.052.2.3 PSSM.

For a protein sequence P, PSSM of P can be computed by PSI-BLAST software, which contains the evolutionary information of P. The matrix is as follows. Additionally, PSSM can be used in another way [43], which calculates pairwise similarity based on both PSSM and PSFM.

(12) $$\mathrm{PSSM}=\left(\begin{array}{cccc} a_{1,1} & a_{1,2} & \cdots & a_{1,20}\\ a_{2,1} & a_{2,2} & \cdots & a_{2,20}\\ \vdots & \vdots & \ddots & \vdots \\ a_{L,1} & a_{L,2} & \ldots & a_{L,20} \end{array}\right)\;$$

where $a_{i,j}$ indicates the score of the *i*th amino acid in P evolving to the *j*th amino acid in the 20 standard amino acids. Next, we used the following formula to convert it into a value between 0 and 1:

(13) $$A_{i,j}=\frac{1}{1+e^{-a_{i,j}}}\;$$

We calculated the average of each column of PSSM by Equation (14) and obtained 20 features. The feature vector is short for *F_pssm_*_-20_:

(14) $$F_{pssm-20}=\left\{\frac{\sum_{i=1}^{L}A_{i,j}}{L}|j=1,2\cdots 20\right\}\;$$

The above 20 features are extracted from the evolutionary information of a sequence, but there is no correlation between amino acids in the sequence. In order to overcome the shortcoming, Zhang et al. [17] proposed an algorithm based on PSSM and the correlation factor ($b_{\lambda }$) computed by PseAAC algorithm. The features are calculated by the following formulas:

(15) $$F_{pssm-400}=\left\{\frac{\sum_{i=1}^{L-g}M(s,t,i,g)}{L-g}|s,t=1,2,\ldots 20,g=\left|s-t\right|\right\}\;$$

(16) $$M(s,t,i,g)=\frac{\left(A_{i,s}-F_{s})(A_{i+g,t}-F_{t}\right)}{\sqrt{\frac{\sum_{j=1}^{L}{\left(A_{j,s}-F_{s}\right)}^{2}}{L}}\sqrt{\frac{\sum_{j=1}^{L}{\left(A_{j,t}-F_{t}\right)}^{2}}{L}}}\;$$

*F_s_* and *F_t_*, respectively, are the average of the *s*-th column and the *t*-th column of PSSM. There are two points that need to be noted in the algorithm. First, the sequence length should be greater than or equal to 20, otherwise *L*-*g* will be 0 (the denominator in Equation (15) will be 0). Secondly, the value of Equation (15) is 1 when *s* and *t* are equal, therefore the features are invalid for *s* = *t* and should be abandoned. That is to say, Equation (15) will be computed for *s* ≠ *t*. Then 20 features (i.e., when *s* = *t*) will be deleted from all 400 features. Finally, a total of 380 features are obtained. The feature vector is short for *F_pssm_*_-380_.

#### 7.2.3. Secondary Structure

The secondary structure of protein has always been used to predict protein structural classes. Here we used it to recognize the function of proteins. The secondary structure of protein sequences are divided into helix, sheet, and coil, which can be abbreviated as H, E, and C.

PSI-PRED software can be used to extract the secondary structure sequence of a protein sequence, which is a sequence consisting of H, E, and C. We obtained three features by calculating the frequency of H, E and C by Equation (11) [44]. Here *L* is the length of the secondary structure sequence and *T(H)*, *T(E)*, *T(C)* are the number of H, E, C:

(17) $$F_{sss-1-3}=\left\{\frac{T(H)}{L},\frac{T(E)}{L},\frac{T(C)}{L}\right\}\;$$

We attained another three features with positional information computed by Equation (18) [44]. Here $P_{H_{i}}$, $P_{E_{i}}$ and $P_{C_{i}}$ are the position index (starts at 1) of the *i*-th H, E, C in the secondary structure sequence:

(18) $$F_{sss-4-6}=\left\{\frac{\sum_{i=1}^{T(H)}P_{H_{i}}}{L(L-1)},\frac{\sum_{i=1}^{T(E)}P_{E_{i}}}{L(L-1)},\frac{\sum_{i=1}^{T(C)}P_{C_{i}}}{L(L-1)}\right\}\;$$

Another three features can be computed by Equation (19) [44], where *MAX*(*L_H_*), *MAX(L_E_)*, *MAX(L_C_)* are the maximal length of all contiguous segments H, E, and C. For example, for the secondary structure sequence EEEEHHEEHHHCC, *MAX*(*L_H_*) = 3, *MAX*(*L_E_*) = 4 and *MAX*(*L_C_*) = 2:

(19) $$F_{sss-7-9}=\left\{\frac{MAX(L_{H})}{L},\frac{MAX(L_{E})}{L},\frac{MAX(L_{C})}{L}\right\}\;$$

For a secondary structure sequence S, there will be the sequence S_0_ with H and E after removing C from S. S_0_ will be a sequence consisting of *α* and *β* when the consecutive H denoted by *α* and the consecutive E denoted by *β* [45]. Then a transition probability matrix (TPM) can be computed by Equation (20) [46,47,48,49,50]. Here *T*(*αα*), *T*(*αβ*), *T*(*βα*) and *T*(*ββ*) are the number of *αα*, *αβ*, *βα* and *ββ* in the sequence:

(20) $$\mathrm{TPM}=\left(\begin{array}{cc} \frac{T(\alpha \alpha )}{T(\alpha \alpha )+T(\alpha \beta )} & \frac{T(\alpha \beta )}{T(\alpha \alpha )+T(\alpha \beta )}\\ \frac{T(\beta \alpha )}{T(\beta \beta )+T(\beta \alpha )} & \frac{T(\beta \beta )}{T(\beta \beta )+T(\beta \alpha )} \end{array}\right)\;$$

Six features can be extracted in terms of TPM, and the corresponding formula is as follows:

(21) $$F_{sss-10-15}=\left\{\mathrm{TPM}(i,j),\frac{\left(\mathrm{TPM}(1,1)+\mathrm{TPM}(2,1)\right)}{2},\frac{\left(\mathrm{TPM}(1,2)+\mathrm{TPM}(2,2)\right)}{2}|i,j=1,2\right\}\;$$

For a secondary structure sequence S, the SPM for S is shown by Equation (22), where $a_{i,j}$ is the possibility of the *i*th element of S predicted to the *j*-th element of C, H and E (1≤i≤L,1≤j≤3):

(22) $$\mathrm{SPM}=\left(\begin{array}{ccc} a_{1,1} & a_{1,2} & a_{1,3}\\ a_{2,1} & a_{2,2} & a_{2,3}\\ \vdots & \vdots & \vdots \\ a_{L,1} & a_{L,2} & a_{L,3} \end{array}\right)\;$$

Then three features can be obtained by Equation (23). Finally, we obtain a total of 18 features based on the secondary structure of protein sequences:

(23) $$F_{sss-16-18}=\left\{\frac{\sum_{i=1}^{L}a_{i,j}}{L}|j=1,2，3\right\}\;$$

## Figures and Tables

**Table 1:** Results of each feature extraction method with different kernel functions.

Feature Vector	Kernel Function	*Acc*	*Sens*	*Spec*	*F*-*score*	*Mcc*
*F_n_*_-*gram*_	linear	80.836%	85.576%	76.174%	81.579%	61.996%
*F_PseAAC_*	linear	81.259%	81.677%	80.836%	81.210%	62.532%
	Gaussian	84.882%	84.192%	85.560%	84.665%	69.766%
*F_pssm_*_-20_	linear	80.402%	76.262%	85.760%	79.863%	62.378%
	Gaussian	76.823%	62.769%	88.829%	72.222%	53.404%
*F_pssm_*_-380_	linear	82.832%	75.378%	88.886%	80.836%	64.865%
	Gaussian	77.588%	63.533%	93.250%	74.470%	59.732%
*F_sss_*	linear	74.242%	77.585%	70.954%	74.939%	48.636%
	Gaussian	72.950%	72.616%	73.296%	72.716%	45.912%

**Table 2:** Results of feature combination methods with different kernel functions.

Feature Vector	Kernel Function	*Acc*	*Sens*	*Spec*	*F*-*Score*	*Mcc*
*F*_sp_	linear	84.081%	84.373%	83.797%	84.025%	68.167%
	Gaussian	86.149%	85.412%	86.885%	85.957%	72.299%
*F*_pssm_	linear	85.700%	81.505%	89.836%	84.970%	71.621%
	Gaussian	78.526%	62.766%	94.050%	74.346%	59.913%
*F*_psp_	linear	89.722%	88.284%	91.132%	89.506%	79.473%
	Gaussian	86.969%	86.046%	87.882%	86.765%	73.951%
*F*_pspn_	linear	89.923%	88.492%	91.325%	89.706%	79.870%
	Gaussian	85.531%	84.951%	86.110%	85.358%	71.062%

**Table 3:** The accuracy of different *C* and *γ* for *F_psp_* with Gaussian kernel.

	*C* = 2^−5^	*C* = 2^−4^	*C* = 2^−3^	*C* = 2^−2^	*C* = 2^−1^	*C* = 2^0^	*C* = 2^1^	*C* = 2^2^	*C* = 2^3^	*C* = 2^4^	*C* = 2^5^
*γ* = 2^−5^	83.936	84.726	85.769	86.934	88.285	89.244	89.974	90.451	90.252	89.878	89.558
*γ* = 2^−4^	83.604	84.316	85.148	86.451	87.754	88.912	89.558	89.606	89.491	89.371	89.286
*γ* = 2^−3^	76.624	79.972	82.856	85.962	86.626	87.917	88.388	88.484	88.466	88.472	88.472
*γ* = 2^−2^	56.476	61.983	64.523	68.565	73.578	80.817	81.402	81.378	81.378	81.378	81.378
*γ* = 2^−1^	50.407	50.407	50.407	50.570	56.995	67.696	69.259	69.259	69.259	69.259	69.259
*γ* = 2^0^	50.407	50.407	50.407	50.407	50.407	51.433	52.404	52.404	52.404	52.404	52.404
*γ* = 2^1^	50.407	50.407	50.407	50.407	50.407	50.419	50.413	50.413	50.413	50.413	50.413
*γ* = 2^2^	50.407	50.407	50.407	50.407	50.407	50.407	50.407	50.407	50.407	50.407	50.407
*γ* = 2^3^	50.407	50.407	50.407	50.407	50.407	50.407	50.407	50.407	50.407	50.407	50.407
*γ* = 2^4^	50.407	50.407	50.407	50.407	50.407	50.407	50.407	50.407	50.407	50.407	50.407
*γ* = 2^5^	50.407	50.407	50.407	50.407	50.407	50.407	50.407	50.407	50.407	50.407	50.407

**Table 4:** Results of 10 times 10-fold cross-validation.

Times	*Acc*	*Sens*	*Spec*	*F*-*score*	*Mcc*
1	90.748% ± 0.567%	89.181% ± 1.003%	92.287% ± 0.880%	90.538% ± 0.617%	81.533% ± 1.133%
2	90.965% ± 0.348%	89.297% ± 1.090%	92.632% ± 0.650%	90.750% ± 0.375%	81.980% ± 0.657%
3	90.851% ± 0.620%	89.238% ± 0.706%	92.450% ± 0.836%	90.644% ± 0.558%	81.737% ± 1.235%
4	90.954% ± 0.695%	89.343% ± 0.890%	92.539% ± 0.727%	90.743% ± 0.772%	81.942% ± 1.395%
5	90.775% ± 0.646%	89.264% ± 1.327%	92.267% ± 0.804%	90.571% ± 0.689%	81.591% ± 1.269%
6	90.819% ± 0.546%	89.232% ± 0.776%	92.382% ± 1.097%	90.612% ± 0.501%	81.673% ± 1.101%
7	90.813% ± 0.682%	89.120% ± 1.024%	92.462% ± 0.960%	90.592% ± 0.725%	81.660% ± 1.367%
8	90.868% ± 0.619%	89.232% ± 0.964%	92.473% ± 0.580%	90.649% ± 0.727%	81.766% ± 1.238%
9	90.895% ± 0.580%	89.302% ± 0.752%	92.460% ± 0.863%	90.692% ± 0.458%	81.816% ± 1.151%
10	90.688% ± 0.532%	89.134% ± 0.985%	92.224% ± 0.567%	90.478% ± 0.572%	81.407% ± 1.048%

**Table 5:** Results of feature combinations with the ratio of the positives to the negatives 1:9.

Feature Vector	Kernel Function	*Acc*	*Sens*	*Spec*	*F*-*Score*	*Mcc*
*F*_PseAAC_	Gaussian	92.315%	31.948%	98.946%	44.943%	46.357%
*F*_sp_	Gaussian	92.875%	37.385%	98.963%	50.743%	51.529%
*F*_pssm-380_	linear	93.520%	43.640%	98.826%	57.745%	57.433%
*F*_pssm_	linear	93.943%	48.157%	98.781%	60.930%	60.319%
*F*_psp_	linear	94.980%	62.231%	98.572%	70.899%	69.132%
*F*_pspn_	linear	94.966%	62.039%	98.574%	70.782%	68.989%
